# Pharmacological Therapies for the Management of Inflammatory Bone Resorption in Periodontal Disease: A Review of Preclinical Studies

**DOI:** 10.1155/2022/5832009

**Published:** 2022-05-02

**Authors:** Angelica Leticia Reis Pavanelli, Bruna Silva de Menezes, Erica Bianca Barbosa Pereira, Fabio Assuncao de Souza Morais, Joni Augusto Cirelli, Rafael Scaf de Molon

**Affiliations:** ^1^Department of Diagnosis and Surgery, São Paulo State University-UNESP, School of Dentistry, Araraquara SP 14801-930, Brazil; ^2^Department of Clinical Dentistry, Federal University of Rio de Janeiro, Rio de Janeiro RJ 21941-617, Brazil

## Abstract

Periodontitis, a highly prevalent multicausal chronic inflammatory and destructive disease, develops as a result of complex host-parasite interactions. Dysbiotic bacterial biofilm in contact with the gingival tissues initiates a cascade of inflammatory events, mediated and modulated by the host's immune response, which is characterized by increased expression of several inflammatory mediators such as cytokines and chemokines in the connective tissue. If periodontal disease (PD) is left untreated, it results in the destruction of the supporting tissues around the teeth, including periodontal ligament, cementum, and alveolar bone, which lead to a wide range of disabilities and poor quality of life, thus imposing significant burdens. This process depends on the differentiation and activity of osteoclasts, the cells responsible for reabsorbing the bone tissue. Therefore, the inhibition of differentiation or activity of these cells is a promising strategy for controlling bone resorption. Several pharmacological drugs that target osteoclasts and inflammatory cells with immunomodulatory and anti-inflammatory effects, such as bisphosphonates, anti-RANK-L antibody, strontium ranelate, cathepsin inhibitors, curcumin, flavonoids, specialized proresolving mediators, and probiotics, were already described to manage inflammatory bone resorption during experimental PD progression in preclinical studies. Meantime, a growing number of studies have described the beneficial effects of herbal products in inhibiting bone resorption in experimental PD. Therefore, this review summarizes the role of several pharmacological drugs used for PD prevention and treatment and highlights the targeted action of all those drugs with antiresorptive properties. In addition, our review provides a timely and critical appraisal for the scientific rationale use of the antiresorptive and immunomodulatory medications in preclinical studies, which will help to understand the basis for its clinical application.

## 1. Introduction

Periodontal disease (PD), a chronic inflammatory condition of the supporting tissues around the teeth, is characterized by the loss of supporting structures of the tooth, such as gingiva, periodontal ligament, alveolar bone, and cementum [1–3]. This condition leads to an irreversible loss of the dental structures and might result in tooth loss if left untreated [1, 4]. The etiology of PD is multifactorial in which the presence of a dysbiotic biofilm in intimate contact with the gingival margin initiates the inflammatory immune response [3, 5, 6]. Indeed, PD is the sixth most prevalent disease globally [7] and is considered the most important cause of tooth loss in the adult population [8].

**Figure 1:**
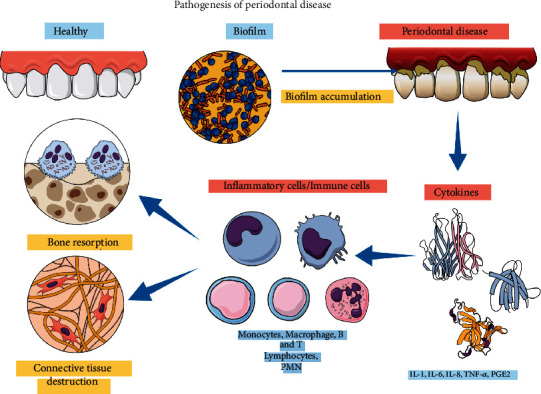
The pathogenesis of PD. The bacteria that compose the dental biofilm trigger the process of local inflammation generated by the increase of cytokines such as IL-1, IL-6, IL-8, TNF-alpha, and PGE2 by the immune cells and inflammatory cells, such as neutrophils and macrophages. Such inflammatory environment ultimately leads to the activation of osteoclasts, the cells responsible to resorb the bone tissue. Consequently, the signs and symptoms of PD (gingival inflammation, epithelial downgrowth, pocket formation, and alveolar bone destruction) occur.

PD is modulated and mediated by the immune host system, which plays an important role in disease severity and progression [6]. During the initiation and progression of PD, environmental conditions (smoking), systemic comorbidities (diabetes mellitus and rheumatoid arthritis), and genetic polymorphisms (IL-1ß) are important aspects that dictate the disease progression [9–11]. In the initiation phase of PD, there is an activation of the inflammatory response, which is characterized by increased gingival crevicular fluid, and an influx of inflammatory cells (leukocytes), especially the polymorphonuclear neutrophils (PMN), that tends to diminish the insult caused by the dysbiotic biofilm [4]. All of these events are protective, and in most patients, the immune system is capable of controlling the disease progression. However, innate and adaptive responses in susceptible patients lead to the aggravation of periodontal tissue destruction. The activation of leucocytes and T cells in the connective tissue leads to the production of multiple inflammatory mediators, degrading enzymes such as matrix metalloproteinases (MMP), and the increased expression of the nuclear factor-kappa B ligand (RANKL), which is the primary activation factor for osteoclasts [12], leading to periodontal inflammation and finally causing the loss of bone supporting tissue (Figure 1) [13–15].

The primary treatment of PD is through scaling and root planning (SRP) to remove the attached biofilm from the root surface. However, removing bacterial biofilm does not imply a return to homeostasis and regeneration of lost tissues [16, 17], and SRP targeting only microorganisms does not accomplish favorable results in all patients [18]. Adjunctive treatments such as systemic local antibiotics, nonsteroidal anti-inflammatory drugs, and low doses of doxycycline have been used as host modulating agents in order to control the progression of PD [19–22]. Despite the clinical benefits of those approaches, their effects are limited in the context of inflammation-induced alveolar bone loss [23]. The major challenge for successfully treating PD is the difficulty in finding a target that can inhibit tissue inflammation and consequently alveolar bone destruction [24]. Therefore, the adjunct use of complementary therapies that are aimed at modulating the destructive events of the immune response has been proposed as a potential therapeutic strategy for PD treatment targeting inflammatory mediators and bone-resorbing osteoclasts.

In recent decades, the use of pharmacological drugs and natural compounds (herbal medicine) aiming to suppress bone destruction during experimental PD in animal models has been extensively reported [25–34]. Interestingly, several studies have shown that inhibition of bone loss can be targeted intervened by innumerous pharmacological drugs, such as alendronate [35–38], OPG-Fc [26], resolvin [39–42], strontium ranelate [27, 43], curcumin [17, 31, 44–47], and cathepsin inhibitors [29, 48]. Therefore, in this review, we comprehensively summarize the roles of several therapeutic drugs during the progression of PD and provide the main findings of each included study leading to the prevention of experimental PD.

In this review, the pharmacological products discussed below are examined through many experiments for their antiosteoclastic activity. The in vivo studies included in this review are based on well-established experimental models of PD, such as ligature-induced bone loss [49–57], lipopolysaccharide (LPS) injections [58–61], and oral inoculation of periodontopathogenic bacteria into the animal mouth [50, 58–60]. Primary methods used to evaluate the inhibition of bone loss were assessed by microcomputed tomography (micro-CT) and histopathological analyses. Oral gavage, palatal injections, and intraperitoneal injections represent the main routes of drug administration in experimental models of periodontitis in rats and mice. Humanized mouse models, subcutaneous bacterial injections, or other animal models were not investigated in this review. We have described the objective, study design, main findings, and conclusions of all the included studies, according to Tables 1–9.

## 2. Cathepsin K Inhibitors

Cathepsin K (CtsK) is a member of the papain superfamily (C1 protein family) of cysteine protease that plays an important role in the innate immune response and osteoclast-mediated bone resorption [24, 62]. It was previously identified as an osteoclast selective protease CtsK [63] abundantly expressed in human osteoclasts, osteoblasts, periodontal ligament cells, osteocytes, and fibroblasts. In the bone tissue, CtsK can cleave the triple helix and the telopeptides from the type I collagen fibers that constitute 90% of the bone organic matrix [64]. In addition, this protease can also activate MMP-9 [65] and degrade type II collagen [66], osteonectin, and osteopontin, thus inhibiting the activity of osteoclasts [64]. It is important to mention that CtsK inhibitors are able to prevent bone resorption without affecting osteoblastic activity. Therefore, the crosstalk between osteoblast and osteoclast is maintained, which is beneficial during bone remodeling [67]. A summary of main study outcomes is described down below (Table 1).

Previous studies have described selective CtsK inhibitors that effectively reduce osteoclast resorption both in vitro and in vivo [68–70]. Furthermore, CtsK has been shown to be an efficient therapeutic strategy in preclinical studies, including inflammatory, metabolic, and autoimmune diseases, such as high fat acid-induced obese mice [71], experimental periodontitis [24, 25, 72], and collagen-induced arthritis (CIA) [72]. However, although CtsK inhibitor has potent inhibitory effects on osteoclast-mediated bone resorption, it has also been associated with some adverse side effects and undesired drug-drug interactions [67, 72, 73]. Odanacatib is an inhibitor of the family member of lysosomal cysteine proteases (cathepsin K inhibitor) involved in the degradation of the demineralized bone matrix; was tested in vitro, in animal models, and in humans; and reached phase III clinical trials [67]. The study was terminated due to an unforeseen increase in cerebrovascular events [74], but odanacatib antifracture efficacy encouraged further studies with new cathepsin inhibitors.

Researches have long held that inflammation and bone breakdown are the two major pathological features of periodontitis and rheumatoid arthritis (RA); consequently, prevention or reduction of these damaging events should be a main therapeutic objective. In this regard, Yue et al. [72] recently investigated the effect of CtsK inhibition on the course of a combined animal model of CIA and experimental PD through oral infection with *P. gingivalis*, a known periodontopathogenic bacterium. The results of this study have demonstrated that inhibition of CtsK by transfection of small interfering RNA (siRNA) resulted in diminished destruction of articular tissue and alveolar bone and decreased the macrophage number and inflammatory cytokine expression in the synovium, suggesting that CtsK inhibition might be implicated as a potential therapeutic strategy in experimental PD and RA [72].

Inhibition of CtsK effectively suppresses autoimmune inflammation of the joints as well as osteoclastic bone resorption in autoimmune arthritis [77]. Pan et al. [75] have used an experimental periodontitis model through oral bacterial inoculation combined with CIA in DBA/1J mice. One week before establishing the combined diseases, animals were treated with CtsK inhibitor BML-244. Alveolar bone resorption and paw swelling were more severe when these two comorbidities were present simultaneously. Furthermore, inhibition of CtsK reduced inflammatory cytokine production and infiltration by dendritic cells and T cells. Consequently, bone loss in PD and RA was abrogated as measured by bone erosion in periodontal lesions and cartilage destruction in knee joints. Inhibition of CtsK also decreased the expression of Toll-like receptor (TLR) 4 and TLR9 in vivo [75].

As previously stated, CtsK also has functions in dendritic cells through the TLR9, which plays a pivotal role in innate immunity recognition of microbial products and in the activation of immune host defense [24, 77]. In this context, Hao et al. [24] evaluated whether inhibition of CtsK would benefit both the immune system and bone system during the progression of bacterial-induced periodontitis in a mouse model. A small molecular inhibitor, odanacatib, was orally administered one week prior to experimental PD establishment. This study demonstrated that oral application of odanacatib decreased the number of osteoclasts, T cells and macrophages, and TLR, thus preventing bone loss and exacerbated immune response during the progression of PD [24]. Moreover, the same study evidenced that lack of cathepsin K inhibited the expression of toll-like receptors 4, 5, and 9 and their downstream cytokine signaling in the gingival epithelial cell, indicating that the innate immune response was abrogated in periodontitis.

Another study evaluated the inhibition of CtsK through adenoassociated virus (AAV) expressing CtsK small hairpin to silence CtsK [25]. Experimental PD was induced by oral gavage with *P. gingivalis*. AAV-sh-CtsK was administered locally into the palatal gingival tissue. The inhibition of CtsK drastically protected the mice from *P. gingivalis*-induced bone loss (>80%) and significantly reduced inflammation in the gingival tissue. The authors suggested that inhibition of CtsK could target both inflammation and bone resorption and efficiently protect against periodontal bone destruction.

Indeed, the use of CtsK inhibitors for the treatment of osteolytic diseases remains promising. However, in contrast to current antiresorptive agents, which target the osteoclast cells, CtsK inhibitors can cause effects in other tissues, as the enzyme not only is present in bone cells but also engages in several other metabolic processes and regulatory pathways. The challenge, then, is to develop more specific inhibitors, which act on the osteolytic activity of the CtsK, without affecting the activity of other enzyme catalytic sites, decreasing the chance of side effects [67, 78].

CtsK activity is regulated by endogenous cysteine proteinase inhibitors, such as cystatin C, which has a high binding affinity to cysteine proteinases [79]. These proteins are capable of inhibiting osteoclastogenesis and bone resorption in vitro and in an ex vivo model [80, 81]. Recently, our group demonstrated that natural inhibitors of cysteine peptidase derived from Citrus sinensis, named phytocystatin CsinCPI-2, was effective in decreasing the gene expression levels of cathepsin K, cathepsin B, IL-1*β*, and TNF-*α*. In addition, CsinCPI-2 significantly inhibited in vivo the activity of TNF-*α* in the blood of rats, previously stimulated by *E. coli* lipopolysaccharide (LPS). These data suggested that CsinCPI-2 has a potential anti-inflammatory effect during bacterial infection in rats [82]. Moreover, we have just showed the positive effects of phytocystatin CsinCPI-2 in the inhibition of bone loss in a mouse model of ligature-induced alveolar bone loss. In this study, it was demonstrated that systemic treatment with CsinCPI-2 significantly reduced inflammatory cell infiltrate, decreased the number of TRAP+ cells, and diminished alveolar bone destruction cause by PD. This treatment also showed downregulation of inflammatory cells expressing CD3, CD45, and MAC387 in the connective tissue. Furthermore, in vitro data demonstrated that CsinCPI-2 inhibited RANKL-induced TRAP+ osteoclast formation in BMM and abrogated RANKL-induced mRNA expression of *Acp5*, *Calcr*, *Ctsk*, and RANKL-induced upregulation of Nfatc1 [76].

## 3. Bisphosphonates

Bisphosphonates, particularly nitrogen-containing ones, such as zoledronate and alendronate, are antiresorptive agents commonly used to treat bone metabolic diseases such as osteoporosis and bone neoplasia, Paget disease, and multiple myeloma. Bisphosphonates inhibit functioning osteoclasts by impairing differentiation, disrupting the cytoskeleton, decreasing intracellular transport, and inducing apoptosis and do so through the inhibition of farnesyl diphosphate synthase in the cholesterol biosynthesis pathway, which prevents prenylation of small guanosine triphosphatase signaling proteins [83–85].

Despite its beneficial effects in inhibiting bone resorption in osteolytic diseases, the use of bisphosphonates, especially intravenous administration of high doses of zoledronate, is associated with adverse side effects. The most significant effect associated with bisphosphonate administration is the osteonecrosis of the jaw (ONJ), a condition defined as an area of exposed bone in the maxillofacial region that does not heal after 8 weeks in patients receiving antiresorptive therapies [86, 87]. Furthermore, atypical fractures are also related to the long-term use of bisphosphonate due to its high maintenance of the drug into the bone tissue. On the other hand, the oral administration of alendronate to treat osteoporosis has shown to have a 0% to 0.4% chance of inducing ONJ [88]. Consequently, several studies have investigated the beneficial effects of alendronate administration to manage experimental periodontitis in rats [35, 37, 89, 90] and PD in clinical trials [91–93]. Some of the described studies are described in table 2.

One of the first studies that have used alendronate as adjunctive therapy to manage experimental PD was conducted by Brunsvold et al. in 1992 [94]. In this study, the authors have induced experimental PD in monkeys by placing a ligature around the mandibular premolars and molars followed by oral inoculation of *P. gingivalis* one week after alendronate administration. Alendronate was administered intravenously for 16 weeks, and clinical and radiographical analyses were performed. The authors demonstrated that 0.05 mg/kg alendronate treatment reduced the progression of PD, suggesting its use to treat PD. Similarly, Moreira et al. [36] have shown that 2.5 mg/kg alendronate administration in rats with experimental PD reduced the activity of osteoclasts and significantly decreased the resorption of the alveolar bone crest. However, after 21 days of treatment, some animals developed signs of ONJ due to the reduced activity of osteoclast. The authors pointed out that using alendronate to treat experimental PD in rats might increase the risk of ONJ development.

The use of alendronate as adjunctive to scaling and root planning (SRP) in rats with induced PD was evaluated by De Almeida et al. [35]. Rats with ligature-induced PD received SRP after ligature removal associated with topical application of alendronate. The animals assigned to receive SRP plus alendronate showed less local inflammation and better tissue repair, associated with higher expression of osteoprotegerin (OPG) immunolabeling, suggesting that the treatment employed might be effective in the treatment of PD in rats. A recent systematic review investigated the potential use of bisphosphonate as an adjuvant to SRP in 13 clinical trials [95]. The results of this systematic literature review demonstrated that locally or systemically administered alendronate reduced probing pocket depth and resulted in a gain of clinical attachment level and improved radiographic assessment. Indeed, bisphosphonate as an adjuvant to SRP may result in clinical benefits in patients with PD. However, the risk to ONJ development after bisphosphonate administration limits their clinical use.

## 4. OPG-Fc and RANKL Inhibitors

**Figure 2:**
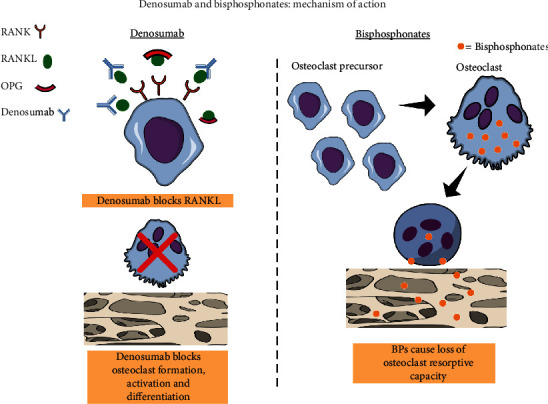
Denosumab acts similarly to OPG, which is RANKL's natural decoy receptor; denosumab binds to RANKL, preventing the binding of RANKL to its receptor, RANK, on the surface of osteoclasts and also on osteoclast precursors. Thus, the RANK signaling pathway is not activated, resulting in impaired osteoclast precursor differentiation and function and possibly osteoclast apoptosis. All these effects lead to inhibition of bone resorption. Bisphosphonates act on osteoclasts, but not on their precursors. Bisphosphonates are internalized into osteoclasts possibly by endocytosis. Subsequently, bisphosphonates inhibit FPP synthase, a key enzyme in the mevalonate signaling pathway. This leads to impaired intracellular protein prenylation impairing osteoclast function and apoptosis. Thus, bone resorption is inhibited.

The discovery of the RANK, RANK ligand (RANKL), and OPG axis has revealed its pivotal role in regulating bone metabolism and created a new field for the study of bone-related diseases [12]. Binding of RANKL to RANK results in the differentiation and maturation of osteoclast precursor cells to activated osteoclasts. Therefore, blocking the interaction between RANK and RANKL is accountable for inhibiting osteoclast differentiation, and it is considered an interesting alternative to inhibit bone loss in osteolytic lesions. Acting as a soluble decoy receptor for RANKL, OPG binds to RANKL and inhibits osteoclast development preventing it from binding to RANK. OPG has been evaluated in preclinical studies of experimental PD as a therapeutic compound for counteracting bone loss (Figure 2).

The pioneering study that has used OPG to treat experimental PD was performed by Teng et al. [96]. Using an oral inoculation infection model with *A. actinomycetemcomitans* in mice, the authors demonstrated that in vivo inhibition of RANKL function with OPG treatment reduces alveolar bone loss and decreases the number of osteoclasts after microbial challenge. These data imply that OPG treatment may thus have therapeutic value to prevent alveolar bone and/or tooth loss in human periodontitis. In this context, Mahamed et al. [97] showed diminished alveolar bone resorption in diabetic mice treated with the RANKL antagonist OPG, which is in agreement with the study of Teng et al. [96]. Using an acute model of ligature-induced bone loss, Jin et al. [26] demonstrated protective effects of OPG-Fc during experimental PD with significant preservation of alveolar bone. Therefore, OPG revealed robust preventive effects on alveolar bone resorption in experimental PD, thus showing a promising therapeutic potential of OPG for PD treatment.

Moreover, an anti-RANKL monoclonal antibody denominated denosumab has been developed and used to treat bone metabolic diseases such as osteoporosis and metastatic bone cancers and other osteolytic bone conditions such as periodontitis and arthritis. Denosumab binds directly to the RANKL to prevent its interaction with RANK on osteoclasts. This binding inhibits osteoclast formation, differentiation, and function [85], thus inhibiting bone resorption. Denosumab does not bind to mouse RANKL; therefore, studies have used an anti-mouse monoclonal RANKL to investigate its potential effects on mice. In this context, Kuritani et al. [98] investigated the effects of systemic administration of anti-RANKL during the progression of ligature-induced bone loss in mice. The study findings showed that anti-RANKL antibody strongly suppressed alveolar bone loss associated with periodontitis. However, similar to bisphosphonates, the potential risk of development of medication-related osteonecrosis of the jaw [99–102] and the use of denosumab or RANKL inhibitors as an adjunctive treatment for PD are not indicated.  Table 3 describes the main study outcomes with RANKL inhibitors.

## 5. Strontium Ranelate (SR)

SR, an antiresorptive compound mainly used for osteoporosis treatment, is a silver-white and soft metallic chemical element. It is placed primarily in areas where mineralization of new bone occurs, such as regions experiencing intramembranous or endochondral ossification [103]. SR is known as a divalent cation that has atomic and ionic properties related to calcium and is also considered as a dual-acting agent that diminishes bone resorption by decreasing osteoclastic activity and stimulating bone formation by proliferation of preosteoblast and secondarily increasing the activity of functional cells and synthesis of bone matrix [104, 105]. This dual-acting mechanism of SR (concomitant antiresorptive and osteoanabolic dual biological activity) represents an advantage over bisphosphonates. Thus, SR is able to increase biomechanical and structural properties of bone, such as mineral density [106]. There are two possible mechanisms of action presented in literature about SR: (1) activating calcium-sensing receptor or another cation-sensing receptor and (2) increasing expression of OPG in addition to decreasing RANKL expression by osteoblasts [107].

One of the first studies investigating the efficacy of SR in preventing bone resorption was made by Marie et al. [108]. This study tested low SR doses on bone loss induced by estrogen deficiency in female rats. Treatment for 60 days with SR resulted in a dose-dependent increase in plasma, urine, and bone strontium concentrations without any deleterious effect on total or skeletal growth. Furthermore, treatment of OVX rats with SR prevented bone loss and bone mineral content was restored to the values in sham rats. Moreover, SR treatment increased the trabecular bone volume up to 30%. On the other hand, two other studies showed that SR administration did not counteract the loss in bone architecture and bone strength in ovariectomized rats [109, 110]. These contradictory findings lead to a deeper investigation of the potential role of SR in other inflammatory diseases such as PD.

In this context, Karakan et al. [27] investigated the effects of SR administration in rats with ligature-induced PD. Three different dosages of SR were used: 300, 625, and 900 mg/kg, and the administration was performed daily by oral gavage. The rats were euthanized 11 days after ligature placement. The results indicated that SR leads to decreased bone loss and reduced osteoclast number. In addition, the number of osteoblast cells was significantly increased after SR treatment. Collectively, the findings of this study suggested that SR at 900 mg/kg might prevent alveolar bone loss in this animal model. Another study conducted by Souza et al. [111] has determined the effect of SR on ligature-induced bone loss in rats. The authors showed that SR prevented periodontal bone loss with concomitant upregulation of heme oxygenase 1 mRNA levels. A recent study also demonstrated the beneficial effects of SR on alveolar bone loss in rats with concomitant PD and estrogen deficiency [43]. The results indicated that SR prevented ligature-induced bone loss in an estrogen-deficiency condition and, to a certain extent, increased trabecular bone area in the presence and absence of periodontal collapse. Furthermore, SR also decreased the expression levels of bone markers, such as RANKL and osteocalcin, appearing to have acted predominantly as an antiresorptive agent. Taken together, the results of these investigations demonstrated that SR plays an important role in inhibiting bone loss in experimental PD (Table 4).

## 6. Biological Therapies

Biological therapies are a novel class of compounds mainly used to treat autoimmune diseases such as rheumatoid arthritis and other chronic inflammatory conditions, i.e., Crohn's disease, ankylosing spondylitis, and ulcerative colitis [18]. Biological therapies include a range of anticytokine agents, including anti-TNF-a, anti-IL-6, anti-IL-1, and T and B cells. These specific agents are monoclonal antibodies that act blocking the activity of cytokines and thus inhibiting the immune-inflammatory response of the host, functioning as an immune suppressant [18]. The use of biological agents to manage experimental PD in animal models has demonstrated potential efficacy for anticytokine therapies in ameliorating bone destruction and reducing inflammatory cell infiltrate [112–114], as described below (Table 5).

### 6.2. Anti-IL-6

A recent study has investigated the effects of systemic administration of anti-IL-6 monoclonal antibodies in the progression of experimental PD in rats [114]. Tocilizumab was intraperitoneally injected immediately after ligature placement, and the animals were sacrificed after 7 and 14 days postoperatively. The results indicated that tocilizumab diminished alveolar bone resorption and attachment loss. Moreover, inflammatory infiltrate was also decreased after treatment. The authors suggested that modulatory therapy with biological agents might be an interesting alternative to inhibit alveolar bone loss, and further studies are warranted to confirm the data.

### 6.3. Anti-TNF-*α*

Tumor necrosis factor-alpha is a key signaling modulator in the pathogenesis of PD, and its upregulation is associated with increased osteoclastogenesis. Thus, investigations targeting TNF-*α* have been evaluated to manage inflammatory bone resorption in animal models. In this context, a recent study evaluated the effects of systemic administration of Etanercept in mice with concomitant diabetes mellitus and periodontitis [115]. Obese diabetic Zucker rats were systemically administered with Etanercept and one week later received ligature to induce experimental PD. Animals were sacrificed after 5 weeks from the baseline. This study indicates that blocking TNF-*α* improves the metabolic status in obese rats with PD and decreases periodontal breakdown associated with diabetes. The same research group also confirmed that anti-TNF-*α* treatment positively impacts the subgingival microbial profile in rats with diabetes and ligature-induced bone loss [116]. Another study investigated anti-TNF-*α* effects with pentoxifylline in an experimental mouse model of chronic antigen-induced arthritis- (AIA-) associated PD [117]. The authors demonstrated that the treatment employed was able to diminish joint inflammation, reduce the levels of TNF-*α* and IL-17, and prevent signs of PD (decreased the number of osteoclasts and recruitment of neutrophils in the connective tissue). In addition, the treatment employed showed the anti-inflammatory and bone protective effects in mice with AIA and concomitant PD. Accordingly, a previous study also demonstrated the positive effects of anti-TNF-*α* on the progression of experimental PD induced by ligature placement by decreasing radiographical bone loss [113]. Finally, Cirelli et al. have used adenoassociated virus vector based on serotype 1 (AAV2/1) to deliver the TNF receptor-immunoglobulin Fc (TNFR:Fc) fusion gene to rats subjected to experimental periodontitis by means of *P. gingivalis* LPS-mediated bone loss [118]. The results showed that AAV2/1-TNFR:Fc administration diminished the levels of several proinflammatory cytokines and osteoclast-like cells in the connective tissue of rats. These data indicate that delivery of AAV2/1-TNFR:Fc might be a feasible approach to modulate PD progression.

## 7. Herbal Medicine

### 7.1. Curcumin

Curcumin is a bioactive compound of turmeric and derived from *Curcuma longa*, a tropical plant native to Southeast Asia [119]. It is a yellow hydrophobic polyphenol composed of three curcuminoids, and it is largely used in dietary spice. It has been reported that curcumin has a variety of biological activities, including osteoimmune modulatory properties and anti-inflammatory, antioxidant, antiangiogenic, and antibacterial effects with the capacity to modulate the innate immune host response [31, 46, 120–123]. Due to the innumerous beneficial effects described in the literature with the use of curcumin to treat experimental PD, natural or chemically modified curcumin has been suggested as an interesting therapeutic approach to managing inflammatory bone resorption [30, 31, 45, 46, 120–123]. Nevertheless, different variables, such as diverse dosages (in vitro and in vivo), vehicle used, and administration route (intraperitoneally, intravenously, and orally), have also been described in the literature [30, 31, 46, 121, 123, 124].

Many investigations have been carried out to evaluate curcumin effects during the progression of experimental PD in murine [30, 31, 45, 46, 120–124] (Table 6). Recently, Pimentel et al. [125] assessed the impact of curcumin (100 mg/kg) on the progression of experimental PD in diabetic rats. The PD model was induced by placing cotton ligatures around the first mandibular molar and in the second maxillary molar. An injection of streptozotocin was intraperitoneally administered in the animals to induce experimental diabetes. Curcumin was administered daily by oral gavage for 30 days. The results indicated that natural curcumin reduces alveolar bone loss and favorably modulates the osteoimmune inflammatory process during disease progression. Interestingly, Zambrano et al. [31] investigate the local administration of curcumin-loaded nanoparticles in an experimental PD model. A model of *Escherichia coli* bacterial lipopolysaccharide (LPS) injection was used to induce PD. The curcumin nanoparticles were locally injected, 2 times per week for four weeks, in the palatal mucosa around the first maxillary molar. Radiographical analysis (micro-CT) showed significant reduction in the loss of alveolar bone caused by LPS in the animals treated with curcumin nanoparticles. A previous study [126] using the silk ligature model of PD in rats demonstrated the potent capacity of oral administration of curcumin (100 mg/kg/day) for 30 days to inhibit bone resorption, which is in agreement with the above-reported studies [31, 125].

Previous studies have used different strategies to enhance the clinical application of curcumin to treat experimental PD. Indeed, chemically modified compounds have been developed to increase their clinical efficacy, which resulted in greater bioavailability maintaining its biological and safety properties [46, 120, 121, 128]. de Almeida Brandao et al. [120] evaluated the effects of a modified curcumin so-called CMC22.4 that is a novel bis-dimethoxy-4-phenylaminocarbonyl curcuminoid. In this study, rats underwent experimental PD using direct microinjections of *Escherichia coli* bacterial LPS into the gingival tissue around the first maxillary molars three times per week. Curcumin was administered daily by oral gavage immediately after LPS injection and continued for the whole experimental period of 28 days. The outcomes showed that CMC2.24 inhibited bone loss, inflammation, and osteoclastogenesis in the LPS-induced periodontitis model even at a low dosage (1 mg/kg/day), suggesting that this compound is more effective than previously documented. Curylofo-Zotti et al. [46] also investigated the effects of CMC2.24 in a model of LPS-induced PD. Similar to the study mentioned above [120], the authors showed that oral administration of curcumin CMC2.24 (30 mg/kg/day) significantly inhibited inflammatory infiltrate in the gingival tissue, decreased the number of osteoclasts, and abrogates bone resorption, pointing to an interesting potential of CMC2.24 in preventing bone resorption in an inflammatory model of PD. Similarly, Elburki et al. [127] showed that oral gavage with CMC2.24 (30 mg/kg/day) also reduced inflammation-mediated connective tissue breakdown in rats with diabetes (induced by intravenous injection of streptozotocin) and PD (induced by *E. coli* LPS injections) and prevented hyperglycemia-induced tissue destruction. CMC2.24 was also able to attenuate the severity of inflammation and bone loss in the periodontal tissues, acting as a potential therapeutic inhibitor of bone resorption in inflammatory conditions. These findings parallel previous observations by the same research group [121] that demonstrated the positive effects of CMC2.24 in inhibiting bone resorption during LPS-induced experimental PD in rats.

Taken together, several studies have demonstrated the beneficial effects of natural curcumin or chemically modified curcumin to treat experimental PD without adverse side effects. Nevertheless, it is important to bear in mind that the differences in dosages used in the studies, the low absorption rate, reduced half-life, and rapid systemic elimination [129] might limit its clinical use to treat PD in humans.

### 7.2. Chalcones

Chalcone is a medicinal plant that has been conventionally used in Brazilian medicine to treat bleeding gums [130]. It is a phenolic compound extracted from the *Myracrodruon urundeuva* (Engl.). This compound presents analgesic and anti-inflammatory properties as evidenced by previous studies in experimental models of inflammation [130, 131]. Moreover, antioxidant, antimicrobial, and antiresorptive properties were previously described during inflammatory conditions, including RA and inflammatory bowel diseases [132, 133]. Therefore, based on the assumption that chalcone presents beneficial properties in inflammation, previous studies have investigated its potential therapeutic effects during experimental periodontitis in rats.

In a study of ligature-induced periodontal bone loss in rats, Botelho et al. [134] assessed the effects of a gel containing chalcones during the progression of PD. Rats underwent nylon ligature placement around the second maxillary molars and received immediately after its placement the chalcone gel (600 *μ*g/g gel) topically applied to the gingival tissues three times per day during the entire experimental period (11 days). The results showed that chalcone gel prevents alveolar bone resorption in the conditions studied and presented with anti-inflammatory and antimicrobial effects during the course of PD.

More recently, Fernandes et al. [47] evaluated the effects of chalcone T4 during the progression of experimental PD. In this study, PD was induced by placing a cotton ligature around the first mandibular molar. Chalcone T4 was systemically administered daily by intragastric gavage (5 and 50 mg/kg) starting on the same day of ligature placement. After 15 days of treatment, the animals were sacrificed, and measurements of radiographical, histological, and molecular analyses were performed. The data indicate that 5 mg/kg of chalcone T4 decreased bone resorption and cellular infiltrate in the connective tissue. Moreover, in vitro data demonstrated that this treatment resulted in a reduced number of osteoclasts and resorption area in raw 267.4 cells. As a proof-of-concept study, the data suggested the potential effect of chalcone T4 as an adjuvant for experimental PD treatment. More studies are warranted to investigate dose response, the effects in different inflammatory models, and the factors that might influence its bioavailability, to better comprehend the pharmacokinetics and pharmacodynamic behavior of chalcone T4 [47].

### 7.3. Flavonoids

In an attempt to pursue natural products with pharmacokinetic, anti-inflammatory, antioxidant, and immunomodulatory effects, growing attention has been dedicated to searching phenolic compounds that might have protective effects on bone and connective tissue [135]. Flavonoids, a group of polyphenolic compounds found in many plants (soybean, olive), fruits (orange peel), vegetables, seeds and beverages, have been suggested as a possible alternative to treat inflammatory bone resorption due to its wide range of biological properties and activities [136]. Therefore, the dietary intake of natural ingredients, including innumerous flavonoids, might be beneficial for bone tissues and can prevent PD progression and severity in different animal models of periodontitis. In this context, many studies have used different types of flavonoids to prevent and treat experimental periodontitis with beneficial effects on the alveolar bone tissue without adverse effects [32–34, 135, 137–142].

Genistein, an isoflavone found in soybean, attenuates alveolar bone loss in a rat model of ligature-induced periodontitis [139]. It has also been reported that genistein inhibits bone loss in ovariectomized (OVX) mice, pointing to an important role in preventing experimental postmenopausal osteoporosis [143]. Taxifolin is a flavanone with potent antioxidant properties that has been shown to stimulate osteoblast differentiation and suppress osteoclastogenesis in vitro [144]. Recently, Lektemur Alpan et al. [142] demonstrated that taxifolin attenuates inflammatory bone resorption in a model of ligature-induced bone loss in rats, decreases inflammatory infiltrate, and improves alveolar bone formation. In an experimental model of LPS-induced inflammatory bone loss, the administration of the flavonoids nobiletin and tangeretin was able to suppress LPS-induced osteoclast formation and bone loss. Furthermore, both flavonoids inhibited osteoclastogenesis in RAW264.7 macrophages [145]. Similarly, the effect of a flavonoid from the bergamot juice could inhibit bone loss and decrease gingival inflammation markers in a rat model of LPS-induced PD [140]. Huang et al. [141] evaluated the effects of myricetin, a naturally occurring flavonoid compound, in an experimental OVX mouse PD model. Systemic administration of myricetin prevented bone loss and enhanced alveolar crest height in vivo, and attenuated osteoclast formation and bone resorption in vitro [141] (Table 7).

Quercetin is an abundant flavonol-type flavonoid that has been associated with innumerous beneficial effects regarding the inflammatory process and immune functions [146–148]. The effects of quercetin on the progression of experimental PD were evaluated by Cheng et al. [138]. Utilizing a model of ligature-induced bone loss, the authors demonstrated decreased alveolar bone loss and reduced inflammatory cell infiltrate in the connective tissue of rats that have received systemic administration of quercetin. Moreover, in vitro data demonstrated that quercetin diminished LPS-induced osteoclast formation, suggesting that it might possess an ameliorative effect during PD progression [138]. Recently, it was demonstrated that a citrus flavonoid—eriocitrin and eriodictyol—diminished inflammatory cell infiltration in the connective tissue of rats with induced PD by means of LPS-injections suggesting that a diet supplemented with flavonoids might enhance local immunity and host defense [137]. Finally, other studies showed beneficial effects of hesperidin [34], luteolin [32], and oleuropein [135] on alveolar bone loss and inflammation in a rat model of ligature-induced PD indicating that flavonoids might be an interesting candidate for modulating inflammatory disease.

### 7.4. Colchicine

Colchicine, a natural compound extracted from *Colchicum autumnale*, possesses innumerous pharmacological properties, such as anti-inflammatory, antioxidant, antimitotic, and antiresorptive, that has been used to treat a variety of inflammatory diseases [149, 150]. The anti-inflammatory and antioxidant effects of colchicine rely on the inhibition of adhesion, mobilization, and chemotaxis of neutrophils and by the disruption of inflammasome activity (NALP3) and IL-1𝛽 secretion. A previous study has shown that colchicine inhibits bone resorption by preventing the release of lysosomal enzymes and blocking osteoclast activity. In this context, Aral et al. investigated the effects of colchicine on cytokine production, apoptosis, alveolar bone loss, and oxidative stress in rats with ligature-induced experimental periodontitis [151]. The animals received two different dosages of colchicine (30 and 100 *μ*g/kg/day) immediately after ligature placement and were sacrificed 11 days after initial treatment. The results showed that colchicine treatment (both dosages) significantly decreased the expression of IL-1*β*, IL-8, and RANKL; RANKL/OPG ratio; total oxidative stress level; and bone volume ratio and increased total antioxidant suggesting that colchicine has prophylactic potential to prevent the progression of bone loss through anti-inflammatory and antiresorptive properties.

## 8. Specialized Proresolving Mediators (SPM)

**Figure 3:**
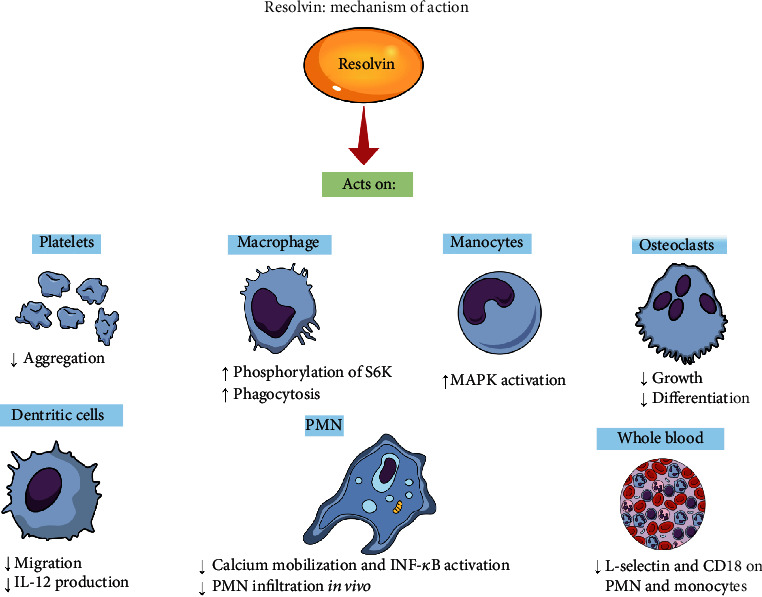
Resolvins (RvE1) act to reduce ADP-stimulated platelet aggregation. In macrophages, RvE1 increases S6K (ribosomal protein S6 kinase) phosphorylation and phagocytosis. In monocytes, MAPK (mitogen-activated protein kinase) activation occurs. RvE1 acts on osteoclasts decreasing their growth and differentiation. In dendritic cells, there is a decrease in their migration and in the production of IL-12. In vitro, RvE1 reduced calcium mobilization and activation of NF-kB, and in vivo, there was a lower infiltration of PMN (polymorphonuclear cell/neutrophil). In the blood, there is a decrease in L-selectin and CD18 in PMN and monocytes.

Current key discoveries in the mechanisms of inflammation during PD initiation and progression encouraged the search for new treatment alternatives for PD using proresolving mediators. Resolution of inflammation comprises active biochemical programs that allow inflamed tissue to return to homeostasis [152, 153]. SPMs are a novel family of oxylipids mediators, including resolvins, maresins, lipoxins, and protectins, derived from omega-3 polyunsaturated fatty acid (PUFA), which regulate the inflammatory process without immunosuppression [7]. The SPMs function in inflammation termination by activating specific mechanisms to restore tissue homeostasis [152, 153]. Briefly, they selectively inhibit leukocyte recruitment, activate macrophage phagocytosis of microorganisms, stimulate infiltration of monocytes, and stimulate the expression of molecules involved in antimicrobial defense [154]. Such SPMs promote tissue repair, eliminate bacteria, increase the host defense, and impact the responses of adaptive immune cells (Figure 3) [39]. The E-series resolvins (RvE1) are biosynthesized from the eicosapentaenoic acid (EPA), and it is considered a stereoselective agonist that interacts with two identified G protein-coupled receptors: BLT1 (expressed on neutrophils) and chemerin receptor 23 (chemR23) expressed on macrophages, monocytes, dendritic cells, and osteoblasts [155, 156]. RvE1 interacts with BLT1 or chemR23 to inhibit leukocyte infiltration and cytokine production, thus promoting the resolution of inflammation [154].

SPMs show significant effectiveness in treating inflammatory conditions including inflammatory pain [157], experimental PD [40, 158, 159], and bone preservation [42]. Furthermore, it has been reported that SPM attenuates atherosclerotic plaque formation in diet- and inflammation-induced atherogenesis [160]. Gao et al. [42] showed that transgenic mice overexpressing the human chemR23 were able to diminish the destruction of the alveolar bone induced by ligature placement. Moreover, local RvE1 treatment accelerated the regeneration of bone defects in a craniotomy model. Taken together, RvE1 modulates osteoclast differentiation and bone remodeling, rescuing OPG production and restoring a favorable RANKL/OPG ratio [42]. This data agrees with the previous report that evaluated the impact of RvE1 on bone remodeling in mice, using a calvaria osteolytic model with or without systemic administration of RvE1 [161]. The data demonstrated that RvE1 reduced bone resorption and osteoclastogenesis. RvE1 also negatively regulated osteoclast differentiation, which resulted in a reduction in inflammatory bone resorption, suggesting that RvE1 may be a therapeutic potential for treating inflammatory diseases [161]. Lee et al. [159] also demonstrated that topical application of RvE1 downregulated bone loss induced by ligature placement and decreased the inflammatory process and the number and size of osteoclasts in rats. In addition, RvE1 induced changes in the composition of the local microbiota suggesting the modulation of local inflammation has an important role in forming the subgingival microbiota composition [159].

Hasturk et al. demonstrated that topical application RvE1 was able to prevent initiation and progression of experimental PD and even induce the regeneration of periodontal tissues (alveolar bone, periodontal ligament, and cement) in a rabbit model of ligature-induced bone loss [40, 158]. RvE1 downregulated the progression of PD by decreasing proinflammatory mediators and reducing inflammatory bone loss. Furthermore, RvE1 is able to enhance the clearance of PD-associated bacteria [40, 158]. These outcomes suggest that PD-associated bacteria actively direct the protective bactericidal immune response into a dysfunctional state, which may be reversed by SPMs. The established protective action of SPM aiming in promoting the resolution of inflammation in innumerous animal models of PD makes them an interesting alternative to treat PD [7, 162].  Table 8 describes the primary findings of the selected studies.

## 9. Probiotics

The manipulation of the intestinal microbiota through probiotics has been proposed to alter bone remodeling during the course of PD both in preclinical studies and in randomized clinical trials. The rationale for this approach is based on the concept that bone health is affected by changes in the intestinal microbiota and therefore, strategies to induce beneficial effects through nutritional supplementation with probiotics have been evidenced. The term probiotics were introduced by Lilly and Stillwell in 1965 [163]. Probiotics are live microorganisms that, when administered in adequate amounts, confer beneficial effects on the host's health. They repopulate beneficial bacteria, which can help kill pathogenic bacteria and fight infection. Orally administered probiotics can benefit oral health by preventing microbiota growth or modulating mucosal immunity in the oral cavity [164]. Probiotics can help prevent and treat PD through several mechanisms, including direct interaction, competitive exclusion, and modulation of the host's immune response. Studies show that the treatment strategies conferred by probiotics against PD occur mainly by inhibiting specific pathogens or altering the host's immune response [165] (Table 9).

Several studies have been published using probiotics for the treatment of experimental PD. Moraes et al. investigated the effects of *L. reuteri* administration during the development of induced PD in rats [166]. The results showed that treatment with probiotics increased the percentage of bone volume and the thickness and number of trabeculae and decreased bone porosity and trabecular separation. Cardoso et al. evaluated the effects of systemic administration of the probiotic *Bifidobacterium animalis* HN019 on ligature-induced periodontitis in rats with experimental RA [167]. Probiotic treatment in animals with experimental arthritis and PD reduced alveolar bone loss, TNF-*α*, and IL-6 levels and increased IL-17 levels compared to those without probiotics. Furthermore, there was a decrease in the levels of anticitrullinated protein antibodies in animals with experimental RA. Ricoldi et al. [168] and Oliveira et al. [169] found similar results using HN019 to treat experimental PD, showing reductions in alveolar bone resorption and connective tissue attachment loss. These results were also observed using different strains of probiotics, including *Lactobacillus rhamnosus* [170], *Lactobacillus brevis* CD2 [171], and *Lactobacillus gasseri* SBT2055 [172].

Some limitations associated with the use of probiotic therapy (difficulty of exogenously administered bacteria in remaining in the oral environment) have stimulated the search for other strategies capable of manipulating the ecology of the oral biofilm [174]. An interesting approach concerns the nutritional stimulation of beneficial native bacteria to promote oral health. Prebiotics favor changes in microbial composition or activity, aiming to stimulate the growth of health-promoting bacteria in the resident intestinal microbiota, which provides local and systemic benefits for the host's health. By definition, prebiotics are selectively fermented ingredients that allow specific changes, either in the composition and/or activity of the gastrointestinal tract microflora, that confer benefits to the host, well-being, and health. They are substances not digested by enzymes, salts, and acids produced by the body. Currently, only oligosaccharides (fructooligosaccharides and galactooligosaccharides) can be called prebiotics. Their mechanism of action occurs through the following: (a) improvement in the growth of resident commensal intestinal bacteria, particularly *bifidobacteria* and *lactobacilli*; (b) they exert a direct effect on the host by stimulating the expression of IL-10 and INF-g, increased secretion of immunoglobulin (IgA), and modulation of inflammatory responses in pathogens [174].

Prebiotics and probiotics often work synergistically and, when combined in the same product, are known as symbiotics. Symbiotics contain both probiotic and prebiotic components. The rationale for such products is that the combination increases the survival of probiotic bacteria in the passage through the proximal region of the gastrointestinal tract, improving colonization of the probiotic in the large intestine, stimulating the effect on the growth of endogenous flora. The main prebiotics evaluated in humans are fructans and galactans. Mannan oligosaccharides (MOS) are also gaining importance. Levi et al. [173] performed a preclinical study in rats demonstrating that animals with ligature-induced PD showed changes in intestinal morphology compared to animals without the disease, confirming the possible relationship between oral and intestinal dysbiosis. When animals with experimental PD were treated with MOS, the intestinal morphology became more similar to that of animals without disease, demonstrating prebiotics' protective role in the intestinal environment under conditions of oral dysbiosis. Furthermore, animals with PD and MOS had less severe PD than those not treated with MOS. In fact, recent scientific evidence suggests that manipulating the microbiota through prebiotics and probiotics confers health benefits on the host through different mechanisms, improving periodontal health and other common skeletal diseases such as arthritis and osteoporosis.

## 10. Vitamins

### 10.1. Vitamin C

Vitamin C has powerful antioxidant properties and has been the focus of several investigations to manage inflammatory diseases, including PD [175]. Deficiency in the levels of systemic vitamin C might affect the gingival and connective tissue increasing the expression of inflammatory cells and impairing collagen formation, thus worsening the severity of periodontitis [176, 177]. A study conducted by Akman et al. evaluated the therapeutic effect of vitamin C on alveolar bone loss in rats with ligature-induced experimental periodontitis [178]. The ligatures were maintained for 5 weeks to induce periodontal breakdown, and then, they were removed. Treatments with vitamin C or vitamin C plus alpha lipoic acid (ALA—50 mg/kg) were initiated immediately after ligature removal with a single intragastric dose for 15 days. Levels of bone alkaline phosphatase and myeloperoxidase activity were measured in the gingival tissues, and expressions of RANKL and bone density were determined histologically. The results indicated that vitamin C and ALA inhibit inflammatory bone resorption and osteoclast activation suggesting its beneficial improvements in osteoclast-mediated bone resorption [178].

### 10.2. Vitamin B

Previously published studies on the effects of food and nutrients with antioxidant and anti-inflammatory activities have constantly been linked to improvements in the periodontal status in animal models [179] and also in patients [180] when treated with vitamin B. Vitamin B complex, a class of water-soluble vitamins, play pivotal functions in cell metabolism [179]. The vitamin B complex includes eight different vitamins which differ in their chemical composition and pharmacological properties [181]. Studies have shown that vitamin B complex is important in soft wound healing and gingival health, and some studies have indicated that vitamin B12 [182], vitamin B9 [183], vitamins B1, B2, B3, B5, B6, and B7 reduced the periodontal destruction and tooth mobility [184]. Recently, Akpınar et al. investigated the effects of vitamin B complex supplementation on the progression of experimental periodontitis in rats. Daily systemic administration of vitamin B by oral gavage was initiated immediately after ligature placement and followed by 11 days. Then, animals were sacrificed and bone tissue samples were collected for histomorphometric evaluation. The authors showed that vitamin B administration increased osteoblast activity, diminished osteoclast numbers, and reduced alveolar bone loss in rat with experimental PD, suggesting beneficial effects of vitamin B complex on the bone tissue.

### 10.3. Vitamins D and K

Vitamin D receptor has been found on many immune cells, such as macrophages, dendritic cells, and T and B cells [185]. Additionally, it has been shown that vitamin D inhibits proinflammatory processes by suppressing the overactivity of CD4+ Th1, Th2, and Th17 cells and the production of their related cytokines such as IL-2, IFN-gamma, and TNF-alpha [186, 187]. Vitamin D has also regulatory effects on bone formation markers, such as osteocalcin and osteopontin, and acts as an immune modulator in inflammatory conditions [185]. Vitamin K plays important roles on bone protection, in the proliferation of bone marrow mesenchymal stem cells, in stimulating osteoblast differentiation and inhibiting adipocyte differentiation. In addition, it can protect osteoblasts and reduce apoptosis. Due to its anabolic effects on bone, the effect of vitamins B and K on gingival inflammation and alveolar bone destruction in rats was investigated by Aral et al. [188]. In this study, periodontitis was induced by placing cotton ligatures around the maxillary first molar for 7 days. Then, ligatures were removed, and tooth received scaling and root planning followed by oral gavage with vitamins D and K or a combination of vitamins D and K for 10 days. The results indicated that alveolar bone loss in rats administrated with vitamin D or K did not differ from rats without treatment, suggesting that this approach has no positive effects on alveolar bone and in gingival inflammatory markers.

## 11. Conclusion

This comprehensive review of the literature summarizes the main findings of studies that have used pharmacological drugs to manage experimental PD. The use of modulators of the immune host response or antiresorptive medications offers interesting alternatives to inhibit bone loss and decrease the inflammatory infiltrate in the connective tissue. All those treatments tested can help modulate the host inflammatory response and ameliorate the progression of the experimental disease. As stated earlier, the primary treatment of PD is through a mechanical approach, SRP, to remove the attached biofilm into the tooth and root surface. However, this local treatment does not respond equally well in susceptible patients. Thus, adjunctive therapies that decrease the inflammatory host response play an important role in achieving better clinical outcomes, especially in patients with associated comorbidities, such as diabetes mellitus and rheumatoid arthritis. It is important to bear in mind that some of the included drugs in this review, i.e., bisphosphonate, biological agents, and RANKL and CtsK inhibitors, possess some side effects that might limit their clinical use. Therefore, herbal medicine and supplementation with omega 3 and probiotics have gained growing attention due to its modulatory and antiresorptive activities and the lack of side effects being considered promising alternatives as adjunctive to SRP in susceptible patients.

## Figures and Tables

**Table 1:** Cathepsin K inhibitors.

Studies	Study design	Main outcomes
Yue et al. (2020) [72]	Animals: eighty male DBA/J1 mice (8 weeks old) Disease model: collagen-induced arthritis (CIA) model. Periodontal disease (PD) model with *P. gingivalis infection* Treatment: injections of adenoassociated virus (AAV) transfection in periodontal tissue and knee joint. AAVs 2.5 × 10^−10^ g/ml. Given once every 3 days for 65 days	Inhibition of articular tissue damage and alveolar bone loss, decreased number of macrophages, and expression of inflammatory cytokines in the synovia, due to inhibition of CtsK
Pan et al. (2019) [75]	Animals: twenty 6- to 7-week-old DBA/1 male wild-type mice Disease model: CIA mouse model and PD model with *P. gingivalis* infection Treatment: CtsK-specific inhibitor BML-244 (25.242 mg/kg per week) or dimethyl sulphoxide (DMSO; vehicle)	Reduced expression of inflammatory cytokines and infiltration by dendritic cells and T cells. Bone loss in PD and RA abrogated. Inhibition of CtsK decreased Toll-like receptor (TLR) 4 and TLR9 expression in vivo
Hao et al. (2015) [24]	Animals: seventy-five 8-week-old female wild-type BALB/cJ mice Disease model: bacterial-induced PD model; 100 *μ*l (5 × 10^9^ CFU/ml of *P. gingivalis*; 5 × 10^9^ CFU/ml of *T. denticola* and *T. forsítia*) topical application eight consecutive times Treatment: orally with 3.606 or 0.7212 mg/kg per week (five times lower dose) of ODN in DMSO for 56 days	Decreased number of osteoclasts, T cells and macrophages, and toll-like receptors in vivo; inhibited the expression of TLRs 4, 5, and 9 and their downstream cytokine signaling in the gingival epithelial cell, indicating that the innate immune response was abrogated
Chen et al. (2016) [25]	Animals: twenty-one wild-type female BALB/cJ mice Disease model: PD induced by oral inoculation with *P. gingivalis* Treatment: gingival injections in the upper molar region of AAV-sh-CtsK or AAV-sh-luc-YFP (3 *μ*l) daily for seven consecutive days	Less bone loss and inflammation in the gingival tissue due to CtsK inhibition
Da Ponte Leguizamon et al. (2022) [76]	Animals: twenty-four 8-week-old C57BL/6J male mice Disease model: ligature-induced periodontal disease Treatment: CsinCPI-2 (0.8 *μ*g/g in PBS) for 15 days	Controlled the inflammatory process, inhibited osteoclastogenesis and alveolar bone loss

**Table 2:** Bisphosphonates.

Studies	Study design	Main outcomes
Brunsvold et al. (1992) [94]	Animals: 27 adult cynomolgus monkeys with intact dentitions Disease model: PD induced by ligature placed around the lower premolars and molars, plus oral inoculation of *P. gingivalis* Treatment: alendronate (0.05 mg/kg) for 16 weeks	Decreased the progression of PD as measured by changes in bone density.
Moreira et al. (2014) [36]	Animals: thirty-six 3-month-old Wistar rats Disease model: ligature-induced PD around the upper right second molar Treatment: daily injections of 2.5 mg/kg body weight alendronate for 7 days before and 7, 14, and 21 days after PD induction.	Reduced the activity of osteoclasts and the resorption of the alveolar bone crest. After 21 days of treatment, some animals developed signs of ONJ due to reduced osteoclast activity
De Almeida et al. (2015) [35]	Animals: ninety 3-month-old Wistar rats Disease model: ligature-induced PD around the lower left first molar Treatment: scaling and root planning and/or administration of alendronate (irrigation with 1 ml of 10^−5^ M) for 7, 15, and 30 days	The combination of the two treatments showed less local inflammation and enhanced tissue repair

**Table 3:** OPG-Fc and RANKL inhibitors.

Studies	Study design	Main outcomes
Teng et al. (2000) [96]	Animals: 8-9-week-old female mice Disease model: oral inoculation infection model with *A. actinomycetemcomitans* Treatment: intraperitoneal injections every other day with PBS or OPG-Fc (1 mg/kg) between weeks 4 and 8	Reduced alveolar bone loss, decrease in the number of osteoclasts
Mahamed et al. (2005) [97]	Animals: 200 NOD mice and 18 BALB/c mice 4-6-week-old female. Disease model: NOD mice were injected with STZ to induce hyperglycemia (40-50 mg/kg). Oral inoculation of *A. actinomycetemcomitans* (10 *μ*g/ml) Treatment: intraperitoneal injections with 2.5 *μ*g hu-OPG-Fc/100 *μ*l PBS, 3 times a week for 8 weeks	Treatment of diabetic mice with OPG leads to the inhibition of bone resorption and reduced RANKL expression, and, therefore, OPG may hold therapeutic potential for treatment bone loss in inflammatory conditions
Jin et al. (2007) [26]	Animals: 32 male Sprague-Dawley rats Disease model: ligature-induced PD placed bilaterally between the lower first molars Treatment: human OPG-Fc (10 mg/kg) or vehicle by subcutaneous injection twice weekly for 6 weeks	OPG-Fc suppressed the number of osteoclasts in the alveolar crest. Preservation of alveolar bone volume
Kuritani et al. (2018) [98]	Animals: 8-week-old male C57BL/6j mice Disease model: LPS-induced calvarial bone destruction. Model of experimental PD using ligatures Treatment: administration of saline solution, anti-RANKL antibodies (3 mg/kg), or zoledronate (0.2 mg/kg).	Anti-RANKL antibodies significantly inhibited alveolar bone destruction and tooth root exposure. Zoledronate suppressed alveolar bone destruction

**Table 4:** Strontium ranelate (SR).

Studies	Study design	Main outcomes
Marie et al. (1993) [108]	Animals: 112 3-month-old Sprague-Dawley female rats Disease model: estrogen deficiency-induced bone loss Treatment: 17 beta-estradiol (10 *μ*g/kg/day, sc) or divalent strontium by gavage at a dose of 77, 154, or 308 mg/kg/day or vehicle for 60 days	Prevented bone loss and increased trabecular bone volume
Karakan et al. (2017) [27]	Animals: 40 Wistar rats Disease model: ligature-induced experimental PD placed around the first molars in the right mandible Treatment: strontium in dosages: 300, 625, and 900 mg/kg. Administration by oral gavage for 11 days	Less alveolar bone loss, reduced number of osteoclasts, and increased number of osteoblast cells. Best results at a dosage of 900 mg/kg
Souza et al. (2018) [111]	Animals: 48 male Wistar rats Disease model: ligature-induced PD placed around the upper molars Treatment: oral administration of strontium ranelate (20 or 100 mg/kg) for 7 days	Prevented bone resorption and increased heme oxygenase-1 mRNA levels in gingival tissues
Marins et al. (2020) [43]	Animals: 96 female Wistar rats ovariectomized Disease model: ligature-induced PD in the mandibular first molar Treatment: oral gavage of 625 mg/kg/day strontium ranelate for 10, 20, and 30 days	Inhibited bone loss, increased the area of trabecular bone, affected the expression of bone markers

**Table 5:** Anti-IL-6 and anti-TNF-*α*.

Studies	Study design	Main outcomes
Apolinario Vieira et al. (2021) [114]	Animals: 90 10- to 12-week-old male Wistar SPF rats Disease model: PD induced by cotton ligature placed on the right first molar in the mandible Treatment: systemic administration of tocilizumab (TCZ) intraperitoneally at concentration dosages (2 mg/kg, 4 mg/kg, and 8 mg/kg) for 7 and 14 days	Inhibited alveolar bone resorption and attachment loss, lower expression of inflammatory infiltrate and lower production of Th17 and RANKL-related cytokines.
Grauballe et al. (2015) [115]	Animals: 80 4-week-old obese diabetic male Zucker rats Disease model: PD induced by ligature placement around the maxillary second molars Treatment: anti-TNF-*α* Etanercept injections for 5 weeks	Blocking TNF-*α* improves metabolic state in obese rats with PD and diminishes periodontal tissue destruction associated with diabetes
Grauballe et al. (2017) [116]	Animals: 52 4-week-old male Zucker rats Disease model: 45 obese rats with type II diabetes and 17 lean rats as controls. PD induced by ligatures around the maxillary second molars Treatment: treatment with subcutaneous injections of 0.5 ml of 0.78 mg/ml Etanercept or RAGE (intraperitoneal injections of 0.8 ml of 1.25 g/l ARA) 3 times a week for 5 weeks	Anti-TNF-*α* treatment has a positive impact on the subgingival microbial profile in rats with diabetes and ligature-induced bone loss
Queiroz-Junior et al. (2013) [117]	Animals: 40 C57BL6 male mice of 6 weeks of age Disease model: mice underwent antigen-induced chronic arthritis (AIA) Treatment: intraperitoneal administration of pentoxifylline (50 mg/kg) daily for 14 days	Decreased expression of TNF-*α* and increased the expression of IL-10 in the maxilla of mice. It did not affect the expression of IFN-*γ* and IL-17. Decreased joint inflammation
Oates et al. (2002) [113]	Animals: 6 Macaca fascicularis from 3 to 7 years old Disease model: PD induced by silk ligatures inoculated with *P. gingivalis* in lower premolars, first and second molars Treatment: intrapapillary injections of soluble receptors (blockers), IL-1 and TNF-*α* (6.6 mg), 3 times a week for 6 weeks	Reduced radiographic bone loss

**Table 6:** Curcumin.

Studies	Study design	Main outcomes
Pimentel et al. (2020) [125]	Animals: 100 10-week-old male rats Disease model: diabetes was induced by streptozotocin. PD was induced by ligatures in the lower first molar and in the upper second molar Treatment: curcumin (100 mg/kg) and placebo solutions and insulin administration by gavage for 30 days	Decreased linear bone loss in the molar region. Reduced RANKL/OPG ratio
Zambrano et al. (2018) [31]	Animals: 16 Holtzman rats Disease model: PD induced by injections of 3 *μ*l LPS (10 mg/ml from *E. coli*) into the maxillary tissues Treatment: 3 *μ*l of nanocurcumin was injected contralaterally from the left side into the gingival tissues twice a week.	Inhibition of inflammatory bone resorption and decreased osteoclast count and inflammatory infiltrate; marked attenuation of p38 MAPK and NF-kB activation
Correa et al. (2017) [126]	Animals: 40 Wistar rats Disease model: Periodontitis induced by silk ligatures around the first molars Treatment: administration by gavage of placebo solution, 10 mg/kg resveratrol, 100 mg/kg curcumin, or 10 mg/kg resveratrol plus 100 mg/kg curcumin for 30 days	Diminished bone loss and inflammatory infiltrate for the resveratrol+curcumin group
de Almeida Brandao et al. (2019) [120]	Animals: 35 male albino rats Disease model: PD induced by LPS injections (*E. coli*) in gingival tissues Treatment: oral gavage of chemically modified curcumin (CMC2.24) at doses: 1, 3, 10, and 30 *μ*M for 28 days	Inhibited alveolar bone resorption, osteoclastogenesis, and expression of TNF-*α*, regardless of dosages
Curylofo-Zotti et al. (2018) [46]	Animals: 50 male rats Disease model: PD induced by LPS injections into the gingival tissues in the maxilla three times a week Treatment: 2% CMC, CMC2.24 30 mg/ kg, curcumin 100 mg/kg. Administered by gavage for 15 days	CMC2.24 was able to reduce alveolar bone resorption
Elburki et al. (2017) [127]	Animals: 18 male Sprague-Dawley rats Disease model diabetes induced by intravenous injection of streptozotocin. PD induced by LPS injection into the maxilla Treatment: CMC 2.24 daily administered by oral gavage (30 mg/kg) for 3 weeks	It inhibited alveolar bone loss and local and systemic inflammation
Elburki et al. (2014) [121]	Animals: 11 male Holtzman rats Disease model: PD induced by LPS injection into the gingival tissue in the maxilla three times a week for 14 days. Treatment: daily oral administration of CMC 2.24 (30 mg/kg) for 14 days	Decreased alveolar bone loss, suppressed the inflammatory process, and decreased the expression of matrix metalloproteinases

**Table 7:** Flavonoids.

Studies	Study design	Main outcomes
Lektemur Alpan et al. (2020) [142]	Animals: 32 male Wistar rats. Disease model: PD induced by ligatures in the lower first molars. Treatment: Administration by oral gavage of taxifolin at doses: 1 mg/kg and 10 mg/kg for 29 days.	Reduced alveolar bone loss. High BMP-2, OCN, ALP, and Col 1 expression and lower RANKL immunoexpression
Tominari et al. (2012) [145]	Animals: 6-week-old male mice Disease model: LPS-induced bone loss (25 *μ*g) on days 0, 2, and 4 for 7 days Treatment: flavonoids—nobiletin or tangeretin (30 *μ*M) for 7 days	Both flavonoids suppressed osteoclast formation and bone resorption. Decreased osteoclastogenesis in RAW264.7 macrophages
Gugliandolo et al. (2019) [140]	Animals: 40 male Sprague-Dawley rats Disease model: PD induced by LPS injection (10 *μ*g/*μ*l) in the gingival tissue between the first and second molars Treatment: bergamot juice flavonoids, 20 mg/kg administered by oral gavage for 14 days	Flavonoid improved the inflammatory process in the gingival tissues. Decreased NF-*κ*B activation and proinflammatory cytokine levels
Huang et al. (2016) [141]	Animals: 24 8-week-old ovariectomized female C57BL/6 mice Disease model: ligature-induced PD in maxillary molars Treatment: intraperitoneal injections of low- or high-dose myricetin (2 or 5 mg) every other day for 30 days	In vivo, it suppressed bone loss and increased alveolar crest height In vitro, it inhibited osteoclast formation and bone resorption
Cheng et al. (2010) [138]	Animals: 6-week-old male Sprague-Dawley rats Disease model: ligature-induced PD in the molars of the maxilla and mandible Treatment: quercetin (75 mg/kg) for 5 days. LPS (5 mg/ml) and quercetin plus LPS	Decreased alveolar bone loss and reduced inflammatory cell infiltrate in connective tissue Decreased LPS-induced osteoclast formation in vitro
Carvalho et al. (2021) [137]	Animals: 60 BALB/c 4-week-old male mice Disease model: PD induced by microinjections of LPS on the palatal surface of both first molars Treatment: food supplement of eriocitrin and eriodictyol (25 and 50 mg) for 30 days	Inhibited periodontal inflammation
Kuo et al. (2019) [34]	Animals: 48 male rats Disease model: ligature-induced PD in the upper and lower first second molars Treatment: hesperidin at doses 75 or 150 mg/kg by intragastric gavage for 7 days	Inhibited alveolar bone loss and the production of proinflammatory mediators
Balci Yuce et al. (2019) [32]	Animals: 28 male Wistar rats Disease model: ligature-induced PD around the lower right first molars Treatment: luteolin 50 mg or 100 mg given by oral gavage for 11 days	Decreased bone loss in both groups. Greater number of osteoblast cells and decreased number of inflammatory cells
Taskan et al. (2019) [135]	Animals: 32 female Wistar rats Disease model: ligature-induced PD in the lower right first molar Treatment: administration by oral gavage of oleuropein 12 or 25 mg/kg for 14 days	Decreased alveolar bone loss due to decreased osteoclastic activity, inflammation, and apoptosis and increased osteoblastic activity

**Table 8:** Specialized mediators in proresolution (SPM).

Studies	Study design	Main outcomes
Gao et al. (2013) [42]	Animals: chemR23tg mice Disease model: ligature-induced PD in the upper left second molar; 1 mm craniotomy defect Treatment: RvE1 (100 ng in 20 *μ*l PBS) or vehicle was injected subcutaneously during craniotomy every 2 days	Less destruction of the alveolar bone after ligatures. It accelerated bone defect regeneration in a craniotomy model
Lee et al. (2016) [159]	Animals: 18 6-week-old male Wistar rats Disease model: ligature-induced PD placed on the upper right and left second molars Treatment: topical resolvin E1 at 0.28 mM or 1.4 mM, 3 times a week for 4 weeks	It reversed bone loss and inflammatory gene expression and reduced osteoclast number for both dosages
Hasturk et al. (2006) [40]	Animals: 21 male white rabbits Disease model: ligature-induced PD followed by *P. gingivalis* injection around the second mandibular premolars Treatment: RvE1, 4 *μ*g applied every other day for 6 weeks	Less progression of PD, decreased proinflammatory mediators, and reduced inflammatory bone loss
Hasturk et al. (2007) [158]	Animals: 39 male white rabbits Disease model: ligature-induced PD followed by *P. gingivalis* infection around the second mandibular premolar Treatment: RvE1, 4 *μ*g applied every other day for 6 weeks	Hard and soft tissue regeneration and decreased inflammation in the periodontal tissues

**Table 9:** Probiotics.

Studies	Study design	Main outcomes
Moraes et al. (2020) [166]	Animals: 32 male rats Disease model: ligature-induced PD; ligature and live *L. reuteri*; ligature and dead *L. reuteri*, in the lower first molars Treatment: live or dead *L. reuteri* given orally 30 days before the disease and 14 days after	Increased alveolar bone volume and trabecular number
Cardoso et al. (2020) [167]	Animals: 32 male Wistar rats Disease model: ligature-induced PD and CIA arthritis model Treatment: probiotic (HN019) in deionized water was supplied to animals (1.5 × 10^9^ CFU/ml) for 39 days	Reduced alveolar bone loss and TNF-*α* and IL-6 levels and increased IL-17 levels. Decreased levels of ACPA antibodies
Ricoldi et al. (2017) [168]	Animals: 32 adult male Wistar rats Disease model: ligature-induced PD around the lower right first molars Treatment: 10 ml of 10% skim milk with *B. lactis* HN019 once daily for 15 days	Reduced alveolar bone resorption and attachment loss. Increased expression of anti-inflammatory cytokines and reduced expression of proinflammatory cytokines
Oliveira et al. (2017) [169]	Animals: 32 adult male Wistar rats Disease model: PD induced by cotton ligatures around the lower first molars Treatment: Probiotic HN019 administered topically to the subgingival region of molars on days 0, 3, and 7	Less alveolar bone resorption and attachment loss
Gatej et al. (2018) [170]	Animals: 36 6-week old BALB/c mice Disease model: PD was induced by oral inoculation with *P. gingivalis* Treatment: probiotic Lactobacillus rhamnosus was given by oral gavage before and during disease induction	Reduced bone loss and gingival inflammation
Maekawa and Hajishengallis (2014) [171]	Animals: C57BL male mice Disease model: ligature-induced PD around the upper left second molar Treatment: *L. brevis* CD2 applied topically between the gingiva and the buccal mucosa	Decreased bone loss and lower expression of TNF, IL-1*β*, IL-6, and IL-17A
Kobayashi et al. (2017) [172]	Animals: 36 8-week-old BALB/c mice Disease model: PD induced by injection of *P. gingivalis* in the mandibular molars Treatment: *Lactobacillus gasseri* SBT2055 (LG2055) given by gavage daily for 5 weeks	Reduced alveolar bone loss and decreased TNF-*α* and IL-6 expression in the gingival tissue
Levi et al. (2018) [173]	Animals: 40 male Wistar rats Disease model: ligature-induced PD Treatment: *Mannanoligosaccharide* (MOS) added daily to the food for 30 days prior to PD	Decreased alveolar bone loss and increased bone mineral density. Decreased expression of IL-10 and IFN-*γ* and TNF-*α* genes

## Data Availability

The data used to support the findings of this study are available from the corresponding author upon request.
